# Carbonic anhydrase influences asymmetric sodium and acetate transport across omasum of sheep

**DOI:** 10.5713/ajas.20.0163

**Published:** 2020-06-24

**Authors:** Imtiaz Rabbani, Habib Rehman, Holger Martens, Khalid Abdul Majeed, Muhammad Shahbaz Yousaf, Zia Ur Rehman

**Affiliations:** 1 Department of Physiology, University of Veterinary and Animal Sciences, Lahore, 54000, Pakistan; 2 Institute of Veterinary Physiology, Free University of Berlin, Berlin 14163, Germany; 3 Department of Physiology, University College of Veterinary and Animal Sciences, The Islamia University of Bahawalpur, 63100, Pakistan

**Keywords:** Flux Measurements, Ovine, Short Circuit Current, Ussing Chamber

## Abstract

**Objective:**

Omasum is an important site for the absorption of short chain fatty acids. The major route for the transport of acetate is via sodium hydrogen exchanger (NHE). However, a discrepancy in the symmetry of sodium and acetate transport has been previously reported, the mechanism of which is unclear. In this study, we investigated the possible role of carbonic anhydrase (CA) for this asymmetry.

**Methods:**

Omasal tissues were isolated from healthy sheep (N = 3) and divided into four groups; pH 7.4 and 6.4 alone and in combination with Ethoxzolamide. Electrophysiological measurements were made using Ussing chamber and the electrical measurements were made using computer controlled voltage clamp apparatus. Effect(s) of CA inhibitor on acetate and sodium transport flux rate of Na^22^ and ^14^C-acetate was measured in three different flux time periods. Data were presented as mean±standard deviation and level of significance was ascertained at p≤0.05.

**Results:**

Mucosal to serosal flux of Na (J_ms_Na) was greater than mucosal to serosal flux of acetate (J_ms_Ac) when the pH was decreased from 7.4 to 6.4. However, the addition of CA inhibitor almost completely abolished this discrepancy (J_ms_Na ≈ J_ms_Ac).

**Conclusion:**

The results of the present study suggest that the additional protons required to drive the NHE were provided by the CA enzyme in the isolated omasal epithelium. The findings of this study also suggest that the functions of CA may be exploited for better absorption in omasum.

## INTRODUCTION

Short chain fatty acids (SCFAs) namely acetate, propionate and butyrate are fundamental in providing energy to the ruminants and are products of microbial fermentation of the ingesta under anaerobic milieu. Rumen provides a complex environment for SCFAs production and is the major compartment where the absorption takes place [1]. The transport of SCFAs across ruminal epithelium has been extensively studied in sheep [2], swine and human colon [3]. Mostly, the transport is through carrier-mediated mechanisms that have been shown to occur through apical as well as basolateral membranes [4,5]. SCFAs transport is closely linked to sodium transport through sodium/hydrogen exchanger (NHE) in large intestine of guinea pigs, sheep, ponies and pigs [6,7].

Significant amount of microbial activity also exists in omasum and consequently SCFAs production and absorption takes place in omasum [8]. There is also an outflow of SCFAs from rumen into omasum depending on dry matter intake and consequent absorption in omasum [9]. The carrier-mediated transport of SCFAs in exchange for bicarbonate ions as occurs in rumen [4], is not possible in omasum because omasal epithelia have predominately absorptive bicarbonate function [1]. Transport mechanisms analogous to that of large intestine have been documented, where SCFAs namely acetate and sodium transport occurs via NHE in omasal epithelia of sheep, that is shown to be mutual but asymmetrical [10]. However, the cause of asymmetry in mutual transport of acetate and Na in epithelial transport in omasum remains unclear.

Several molecules have been identified as regulators of different isoforms of NHE, including but not limited to protein kinase A & C [11], insulin [12], tenapanor hydrochloride [13]. In the current study, we have assumed that omasal intraepithelial carbonic anhydrase enzyme (CA) is contributing to asymmetry. The CA in present in many cells both intracellularly and on the surface. It is mainly involved in the reversible conversion of carbon dioxide and water to bicarbonate and protons also maintains acid-base balance [14]. Using acetate as the principal SCFA, mutual asymmetrical interaction between acetate and sodium transport via NHE was described previously [10], and the CA was inhibited to elucidate it as a factor responsible for asymmetry. This study specifically focuses on the possible role of CA in providing additional H^+^ to the NHE and thus causing the asymmetry in the isolated omasal epithelium of sheep.

## MATERIALS AND METHODS

### Animals

Sheep (*Ovis aries*) (N = 3) of almost alike age and weight from both sexes were selected. The animals were given hay *ad libitum* 2 weeks prior to experiment and had free access to water and a lick stone.

### Tissue collections and preparation

Omasal samples were isolated from sheep (N = 3) as previously described by Ali et al [10,15]. Briefly, sheep were killed after stunning at slaughterhouse of Free University of Berlin (FUB), Germany and the fore-stomachs were removed from abdomen within 3 to 4 minutes. Omasum was identified and separated from reticulorumen and abomasum, and was opened by a longitudinal cut down the omasal canal. The inner part was everted and washed with warm phosphate buffer saline (PBS). Eight to ten slices, each of approximately 250 to 300 cm^2^ surface area were sectioned from omasal wall. The mucosal surfaces were stripped from these slices with caution and transported to laboratory in Institute of Veterinary Physiology, FUB, within 25 minutes in warm (38°C) and continuously gassed (95% oxygen, 5% carbon dioxide) PBS. The protocols followed for animal housing and slaughtering adhered to guidelines of FUB, Germany (Ethical approval permit # T0064/99).

### Experimental groups

Experimental conditions were followed as described previously by Ali et al [10] with slight modifications. Selected omasal tissues were randomly divided into four different groups as pH 7.4 (Control) and pH 6.4 alone and in combination with Ethoxzolamide (Sigma, Taufkirchen, Germany).

### Electrical measurements

A 3×3 cm piece of omasal epithelium was used for mounting in Ussing chamber. A control buffer of pH 7.4 was prepared with bicarbonate ions and SCFA concentration of 40 mmol/litre. Control buffer was made as described previously by Rabbani et al [16] with slight modification. Briefly the control buffer constituted of (in milli-moles/litre): NaCl (20), MgCl_2_ (1), CaCl_2_ (1), NaHCO_3_ (25), K_2_HPO_4_ (2), KH_2_PO_4_ (1), Na-acetate (24), Na-propionate (12), Na-butyrate (4), Na-gluconate (20), and Glucose (10). The tissues were bathed with 16 mL of control buffer on each side of chamber at 38°C. All the chambers were continuously gassed with 95% O_2_ and 5% CO_2_. At least 20 minutes were given for the electrophysiological measurements to stabilize. Following equilibration period, omasal tissues were considered viable and included in this study based on conductance (G_t_) <8.0 Ms/cm^2^ and short-circuit current (I_sc_) >1.0 μA/cm^2^ as described by Martens and Gabel [17].

### Flux measurements

Unidirectional flux measurements were made as described previously by Ali et al [10]. Luminal side pH was adjusted to 6.4 or 7.4 using 1 mM/L hydrochloric acid or Tris-hydroxymethyl-aminomethan in respective groups with serosal pH of 7.4 in all chambers irrespective of groups. Omasal tissues were again stabilized under the new experimental conditions for about 25 minutes. Afterwards, 0.1 mM ethoxzolamide was added on both sides of chamber to see effect of CA inhibitor on acetate and sodium transport Flux rate of Na^22^ and ^14^C-acetate was measured in three different flux time periods, each of 30 minutes.

**Figure 1:**
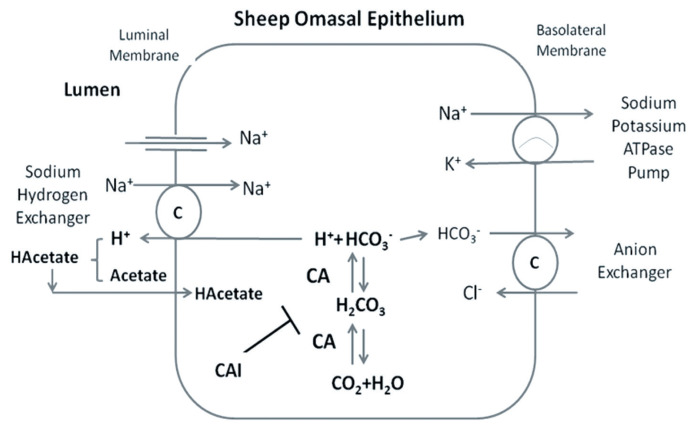
Inhibiton of carbonic anhydrase enzyme (CAI) through ethoxzolamide in sheep omasal epithelia and its link with sodium and acetate transport. C indicates carrier transport ers (Na^+^/H^+^ exchanger and Cl^−^/HCO_3_^−^ exchanger). CA indicates carbonic anhydrase enzyme (Model adapted from [18,19]).

For detection of fluxes of Na and acetate from mucosa to serosa (J_ms_) and serosa to mucosa (J_sm_), 70-kBq ^22^Na and 60-kBq ^14^C-acetate radioactively labelled were used, respectively. After equilibration of tissues, ^14^C-acetate and ^22^Na was added to the radioactively labelled side. A 100 μL sample was taken from radioactively labelled side after 15 minutes (H1) and then at the end of the protocol (H2). Within 30 minutes between these samples, 1 mL samples were taken from the opposite side of radioactively labelled tissue. The volumes taken were replaced by control physiological buffer in each case with adjusted pH. The samples collected from both the radioactively labelled side and opposite side were made in a 5 mL scintillator solution (Zinsser Analytic, Frankfurt, Germany). The solution was properly shaken for measuring the isotopes radioactivity using a β-counter Liquid Scintillation analyzer (Perkin Elmer, Überlingen, Germany). The electrical measurements were made using computer controlled voltage clamp apparatus (Micro clamp, Datentechnik, Aachen, Germany). The resistance of fluid in bridges for sensing Trans-epithelial potential difference (T_PD_) was measured before mounting of tissue and correction applied by computer controlled clamp apparatus. Tissues were kept in short-circuit condition throughout the experiment with the application of bipolar pulse of 100 μA for 0.2 seconds duration. The change in T_PD_, I_sc_, and G_t_ was calculated after every 10 seconds. The data was stored in voltage clamp apparatus computer software for further analysis. The model is depicted in Figure 1.

### Statistics

The data was analyzed using Sigmaplot (Systat software Inc., San Jose, CA, USA, Version 11.0). One way analysis of variance with Tukey’s test was used to compare the data. Data was presented as mean±standard deviation. Level of significance at p≤0.05 was considered significant.

## RESULTS

A decrease in pH of luminal side caused an increase in J_ms_ of acetate and sodium. At pH 7.4, the sodium J_ms_ was 7.23±0.84. A decrease in pH to 6.4 significantly increased the J_ms_ of sodium to 10.60±0.91 (p<0.05). Similar trend of increased was noticed with acetate having J_ms_ 2.76±0.20 at pH 7.4 to 3.79± 0.34. The increase of the acetate was not statistically different. An asymmetrical increase was observed in sodium and acetate transport. The increment of J_ms_ sodium was greater than J_ms_ acetate when the pH was lowered from 7.4 to 6.4 (J_ms_ Na >J_ms_ acetate). There was a net movement of sodium form mucosal to serosal side with J_net_ 4.31±0.57. The flux of acetate in pH 7.4 did not show any net absorption or secretion (J_ms_ 2.76±0.20 vs J_sm_ 2.73±0.35) and the net transport of acetate was (J_net_) 0.02±0.19. This is shown in Tables 1 and 2.

**Figure 2:**
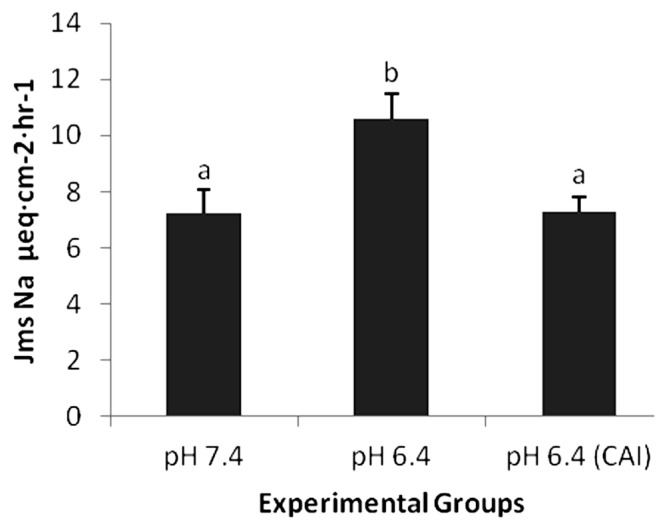
Effect of the addition of carbonic anhydarase inhibitor (CAI) on sodium flux (J_ms_ Na μeq·cm^−2^·h^−1^). The addition of CAI on mucosal side significantly abolishes (p<0.05) the pH dependent asymmetry.

The inhibition of CA enzyme by ethoxzolamide at pH 7.4 significantly decreased J_ms_ of sodium from 7.23±0.84 to 4.21± 0.47 (p<0.05). The decrease was also noticed for acetate with J_ms_ 2.76±0.20 to 2.65±0.24 (p>0.05). The lowering of pH from 7.4 to 6.4 with inhibition of CA through ethoxzolamide increased J_ms_ acetate from 2.76±0.20 to 3.53±0.28 and J_ms_ sodium from 7.23±0.84 to 7.28±0.55. The asymmetry in the increment of J_ms_ Na and acetate (J_ms_ Na>J_ms_ acetate) observed previously is almost absent in the presence of ethoxzolamide, due to CA inhibiton. The obtained data augment the hypothesis that the “missing” protons between uptake of acetate (pH 6.4) and the stimulation of J_ms_ Na are produced by the activity of CA (Figure 2).

## DISCUSSION

Considerable amounts of SCFAs are produced in the rumen and reticulum namely acetate, propionate and butyrate [20]. The role of omasum in this context has not been investigated much and fewer reports [1,8,10] highlight the fermentative and absorptive capability of omasum in context of SCFAs. Furthermore, there is paucity in literature about the moonlighting role of CA in fermentation and absorption of SCFAs in omasum. In the current study, there is very little transport of acetate in omasal epithelia (J_net_ = 0.02±0.19) at pH 7.4 as shown in Table 2 indicating absence of an active transport mechanism for acetate. Similar finding has been shown for butyrate at pH 7.38 where the net flux was 0.1±0.3 μeq/cm^2^/h in distal colon of rats [21]. In isolated colon of pigs, there is an increase in net sodium absorption and bicarbonate accumulation on the luminal side in presence of acetate signifying that acetate may provide an energy source for active sodium transport in colonic epithelium [22]. Similar carrier mechanisms of acetate bicarbonate exchange have been shown in rumen [23], which are unlikely in omasal epithelia because of their absorptive tendency for bicarbonate ions [1].

A decrease in pH to 6.4 from 7.4 considerably increased the J_ms_ acetate to 3.79±0.34 from 2.76±0.20 and J_net_ 1.13±0.42 from 0.02±0.19 (Table 2). There is an increase in protonated form of acetate by decrease in omasal luminal pH, which is lipophilic and easy to pass through the membrane [10]. The decrease in pH from 7.4 to 6.4 of the mucosal side of omasal epithelia increased the J_ms_ sodium and acetate fluxes mutually. Sodium hydrogen exchanger and its interaction with butyrate is studied in cultured ruminal epithelial cells of sheep where intra-epithelial acidification by butyrate is counter-regulated by NHE [24]. A compelling evidence also exists regarding presence of NHE on the luminal wall of the rumen and omasum, which is sensitive to amiloride (inhibitor of NHE) when applied on the mucosal side [17].

Reciprocated interaction has been shown between; propionate or butyrate and sodium transport in proximal colon of rabbits [25], butyrate and sodium transport in distal colon of rat [26], butyrate and sodium transport in gallbladder of guinea pig [27]. Asymmetry in transport fluxes in ovine omasum is reported (10) where J_ms_ of sodium was greater than J_ms_ acetate when pH was lowered from 7.4 to 6.4. This shared but unequal interaction between acetate and sodium transport via NHE is presumably because of the role of CA. The CA uses intra-epithelial carbon dioxide and water to provides protons (H^+^) and bicarbonate (HCO_3_^−^) ions, and H^+^ is exchanged for sodium through NHE [18]. The inhibition of CA decreased the J_ms_ sodium from 10.60±0.91 (pH 6.4) to 7.28±0.55 (pH 6.4 with CA inhibition). This was only slightly increased compared to J_ms_ sodium of 7.23±0.84 at pH 7.4. In sheep omasal epithelium, the H^+^ ion transported to mucosal side through NHE bind with acetate and the protonated SCFA (HSCFA) is formed [17]. HSCFA are lipid soluble and tend to equilibrate through a concentration gradient mechanism from omasal mucosa to intracellular epithelium [21]. The acetate flux J_ms_ also decreased from 3.79±0.34 (pH 6.4) to 3.53±0.28 (pH 6.4 with CA inhibition). The asymmetry observed in the present study and by Ali et al [10] was removed through CA inhibition and statistically non-significant J_ms_ at pH 7.4 and pH 6.4 with CA inhibition for acetate (2.76±0.20 and 3.53±0.28) and sodium (7.23±0.84 and 7.28±0.55) were observed.

## CONCLUSION

It can be deduced that intra-epithelial CA enzyme is responsible for asymmetry in mutual transport of acetate and sodium. The asymmetry was successfully removed by inhibition of CA by ethoxzolamide. This appeared to be the first study exploring the role of CA in SCFAs transport in sheep abomasum. CA may be a potential target for exploitation to improve the absorption of SCFAs in omasum and contribute in better and cost effective production.

## Figures and Tables

**Table 1:** Effects of mucosal and serosal addition of Carbonic anhydrase inhibitor, ethoxzolamide (0.1 mM), on unidirectional sodium flux rates of ovine omasum epithelium

Items	${\text{J}}_{ms}^{Na}$	${\text{J}}_{sm}^{Na}$	${\text{J}}_{net}^{Na}$	N/n

	μeq·cm^−2^·h^−1^			
pH 7.4	7.23±0.84^a^	2. 91±0.72^a^	3.70±0.45^ab^	3/14
pH 7.4 with CAI	4.21±0.47^b^	2.1±0.19^a^	2.25±0.26^b^	3/17
pH 6.4	10.60±0.91^c^	5.41±0.75^b^	4.49±0.71^a^	3/19
pH 6.4 with CAI	7.28±0.55^a^	4.55±0.41^b^	2.73±0.25^ab^	3/16

Data is represented as mean±standard error of the mean.

N, number of animals; n, number of tissues; CAI, carbonic anhydrase inhibitor.

a–c Means with different superscripts within a column differ significantly (p<0.05).

**Table 2:** Effects of mucosal and serosal addition of Carbonic anhydrase inhibitor, ethoxzolamide (0.1 mM), on unidirectional acetate flux rates of ovine omasum epithelium

Items	${\text{J}}_{ms}^{Ac}$	${\text{J}}_{sm}^{Ac}$	${\text{J}}_{net}^{Ac}$	N/n

	μeq·cm^−2^·hr^−1^			
pH 7.4	2.76±0.20^ab^	2.73±0.3	−0.23±0.28^a^	3/14
pH 7.4 with CAI	2.65±0.24^a^	2.83±0.49	−0.19±0.29^a^	3/17
pH 6.4	3.79±0.34^b^	2.66±0.27	1.13±0.42^b^	3/18
pH 6.4 with CAI	3.53±0.28^ab^	3.43±0.24	0.09±0.18^a^	3/16

Data is represented as mean±standard error of the mean.

N, number of animals; n, number of tissues; CAI, carbonic anhydrase inhibitor.

a,b Means with different superscripts within a column differ significantly (p<0.05).
